# Effect of Co Doping on Electrocatalytic Performance of Co-NiS_2_/CoS_2_ Heterostructures

**DOI:** 10.3390/nano11051245

**Published:** 2021-05-08

**Authors:** Zehui Peng, Shuai Lou, Yuan Gao, Lijun Kong, Shancheng Yan, Ka Wang, Haizeng Song

**Affiliations:** 1 School of Geography and Biological Information, Nanjing University of Posts and Telecommunications, Nanjing 210023, China; 1019172222@njupt.edu.cn (Z.P.); 1218063835@njupt.edu.cn (Y.G.); 1020173018@njupt.edu.cn (L.K.); 2 Department of Materials Science and Engineering, University of California, Berkeley, CA 94720, USA; slou@berkeley.edu; 3 Collaborative Innovation Center of Advanced Microstructures, Nanjing University, Nanjing 210093, China; 1217063729@njupt.edu.cn (K.W.); DG1823028@smail.nju.edu.cn (H.S.)

**Keywords:** hydrogen evolution reaction, catalysts, heterostructures, transition metal dichalcogenides

## Abstract

There are abundant water resources in nature, and hydrogen production from electrolyzed water can be one of the main ways to obtain green and sustainable energy. Traditional water electrolysis uses precious metals as catalysts, but it is difficult to apply in massive volumes due to low reserves and high prices. It is still a challenge to develop hydrogen electrocatalysts with excellent performance but low cost to further improve the efficiency of hydrogen production. This article reported a potential candidate, the Co-NiS_2_/CoS_2_ (material is based on NiS_2_, and after Co doping, The NiS_2_/CoS_2_ heterostructure is formed) heterostructures, prepared by hydrothermal method with carbon paper as the substrate. In a 0.5 M sulfuric acid solution, the hydrogen evolution reaction with Co-NiS_2_/CoS_2_ as the electrode showed excellent catalytic performance. When the Co (Cobalt) doping concentration is increased to 27%, the overpotential is −133.3 mV, which is a drop of 81 mV compared with −214.3 mV when it is not doped. The heterostructure formed after doping also has good stability. After 800 CV cycles, the difference in overpotential is only 3 mV. The significant improvement of the catalytic performance can be attributed to the significant changes in the crystal structure and properties of the doped heterostructures, which provide an effective method for efficient electrocatalytic hydrogen production.

## 1. Introduction

Large scale use of pollution-free, green and sustainable energy is the future development trend [1,2,3]. Compared with traditional energy sources, the water produced by hydrogen combustion will not cause any pollution to the atmosphere and water resources [4,5,6,7]. At present, the common hydrogen production methods include photocatalysis and electrocatalysis, etc. [1,3]. Among them, catalysts-assisted water splitting is one of the most promising methods to generate hydrogen [8,9,10]. Precious metals (platinum and palladium based) are common catalysts for water splitting, and the onset potential of the Pt electrode is close to 0 mV [11]. However, due to its scarcity and high price, it is difficult to widely use in industrial hydrogen production [12,13,14,15,16,17,18].

Non-noble transition metal sulfides have attracted extensive attention due to their simple synthesis, low price, good stability and other advantages [19]. The transition metal sulfide catalysts with Fe, Co and Ni have the problems of insufficient active centers and low conductivity, which limit the efficiency of hydrogen production. Co doping can significantly improve the efficiency of electrocatalytic hydrogen evolution, and with the increase in Co doping, the formed heterostructures can show unique properties. Compared with NiS_2_ (Nickel disulfide) nanomaterials, Co-NiS_2_/CoS_2_ heterostructures can promote the rapid transfer of electrons and have strong reducibility [20,21,22]. Looking for the best Co doping ratio to improve the hydrogen production efficiency of transition metal sulfides is of great significance to explore green and sustainable development, but there are few studies on the influence of the Co doping ratio on the hydrogen evolution performance of transition metal sulfides.

In this paper, Co-NiS_2_/CoS_2_ heterostructures were prepared by the hydrothermal method, which showed good catalytic performance in 0.5 M H_2_SO_4_. With the increase in Co content, the catalytic activity is enhanced. When the current density is 10 mA∙cm^−2^, the overpotential changed from −214.3 mV to −133.3 mV with the increase in Co doping ratio, the absolute value of overpotential decreased by 81 mV, and the catalytic activity increased significantly. The relationship between the electrochemical performance and chemical characterization of Co-NiS_2_/CoS_2_ heterostructures with different doping concentrations was explored to find the best doping ratio. Doping a certain proportion of non-noble metal catalyst is an effective way to improve the efficiency of electro catalytic hydrogen production, which is expected to solve the problems of the high cost of traditional noble metal electro catalytic hydrogen production and the low efficiency of non-noble metal hydrogen production.

## 2. Materials and Methods 

### 2.1. Materials and Chemicals 

SC(NH_2_)_2_, NiSO_4_∙6H_2_O used in the experiment was purchased from Shanghai Titan Technology Co., Ltd. (Shanghai, China). Sulfur powder (S) was obtained from Nanjing Chemical Reagent Co., Ltd. (Nanjing, China). Co(NO_3_)_2_∙6H_2_O used in the experiment was purchased from Shanghai Aladdin Biochemical Technology Co., Ltd. (Shanghai, China), while H_2_SO_4_ and C_2_H_5_OH were acquired from Shanghai Titan Technology Co., Ltd. (Shanghai, China). 

### 2.2. Synthesis of Co-NiS_2_/CoS_2_ Heterostructures

**Figure 1:**
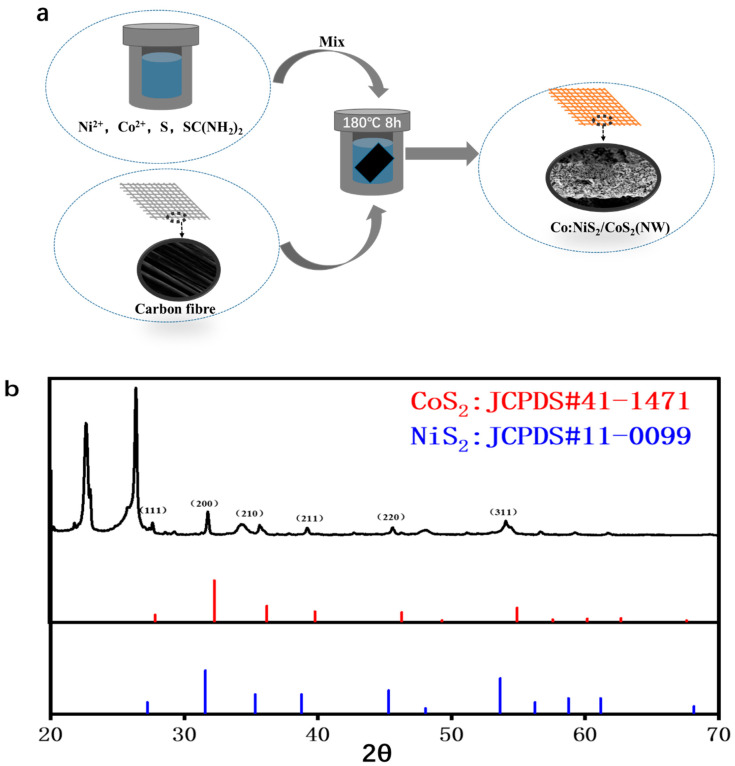
(**a**) Flow chart of hydrothermal preparation of Co-NiS_2_/CoS_2_ heterostructures; (**b**) XRD pattern of Co-NiS_2_/CoS_2_ heterostructures doped with 27% Co.

The preparation of Co-NiS_2_/CoS_2_ heterostructures is shown in Figure 1a. Take the preparation of Co-NiS_2_/CoS_2_ heterostructures with 27% Co doping ratio as an example: the carbon paper (2 cm × 2 cm) is washed with deionized water and ethanol for 15 min, respectively. Co(NO_3_)_2_∙6H_2_O (0.324 mM), NiSO_4_∙6H_2_O (1.2 mM), SC (NH_2_)_2_ (1.8 mM) and 25 mL deionized water are added to 50 mL polytetrafluoroethylene reactor. The reactor is placed on a magnetic stirrer and stirred at a high speed for 15 min to form a uniform and transparent solution. Then, the 0.96 mM sulfur powder is slowly poured into the above reactor during the stirring process, the stirring speed is reduced, and the stirring is continued for 10 min. After the stirring is stopped, the sulfur powder forms a film on the solution. Then, the clean carbon paper is stuck vertically in the reactor, the carbon paper needs to be completely immersed in the solution, and then the reactor is tightened and placed in the 180 °C blast furnace for 8 h. After the reaction, the Co-NiS_2_/CoS_2_ heterostructures condensed on the surface of carbon paper were taken out of the reactor together with carbon paper, and washed twice with alcohol and deionized water. Finally, the residual deionized water on the surface is blown dry with a hot air blower.

### 2.3. Materials Characterization

The crystals Co-NiS_2_/CoS_2_ was analyzed by X-ray diffractometer (XRD) (Bruker Daltonics Inc., Karlsruhe, Germany). The micromorphology of the heterojunction was obtained by scanning electron microscope (FE-SEM; JSM-7000F JEOL Ltd., Tokyo, Japan). Transmission electron microscope (TEM) and Energy dispersive X-ray (EDXA) were taken by JEOL type JEM2100 instrument (JEOL Ltd., Tokyo, Japan). The chemical structure and element valence state of heterostructures were analyzed by X-ray electron spectrometry (XPS, PHI5000 Versaprobe Ulvac-Phi Inc., Kanagawa, Japan). Raman measurements by Horiba LabRAM system (HORIBA, Ltd., Kyoto, Japan).

### 2.4. Electrochemical Measurements

The CHI760E electrochemical analyzer (CH Instruments, Chenhua Co., Shanghai, China) was used to analyze the performance of samples. The test uses a three-electrode system with the sample, platinum and saturated calomel electrode as the working electrode (the loading of the catalyst on the carbon paper, about 11 mg), counter electrode and reference electrode, respectively.

## 3. Results and Discussion

In order to further study the crystal structure of the samples, we measured the X-ray diffraction (XRD). The XRD patterns of Co-NiS_2_/CoS_2_ are shown in Figure 1b. The diffraction peaks at 2θ = 27.2°, 31.6°, 35.3°, 38.8°, 45.3° and 53.6° can be aligned to the NiS_2_ (JCPDS#11-0099) plane at (111), (200), (210), (211), (220), (311) [22,23,24]. There are also some peaks corresponding to CoS_2_ (JCPDS#41-1471) [25,26,27], and bare carbon fiber shows peak located at 26.4° [28]. Compared with pure NiS_2_, these peak positions are shifted by 0.04° after Co doping, which indicates that Co is doped into the NiS_2_ phase [29].

**Figure 2:**
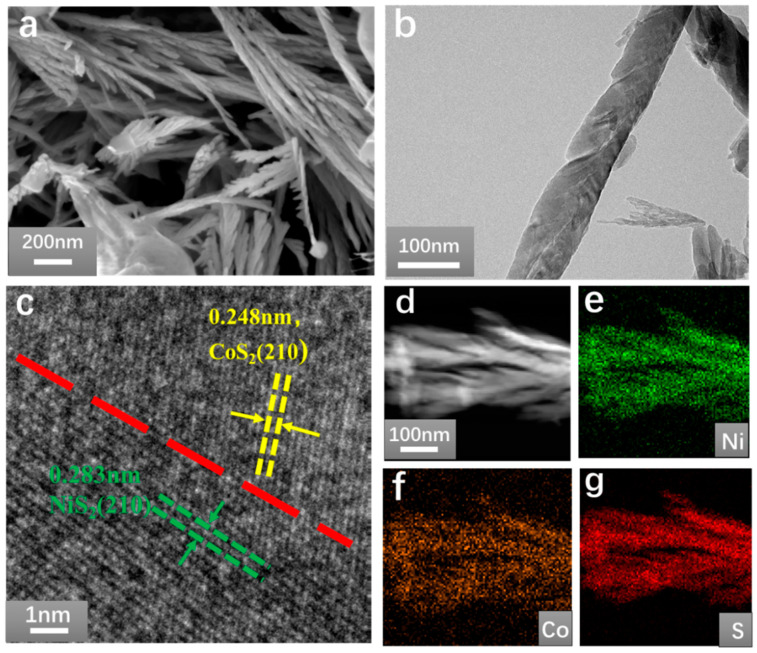
(**a**) SEM images of Co-NiS_2_/CoS_2_ heterostructures; (**b**) TEM image of Co-NiS_2_/CoS_2_ heterostructures; (**c**) HRTEM image of Co-NiS_2_/CoS_2_ heterostructures; (**d**–**g**) STEM image and EDXA elemental mapping of Ni, Co, and S for Co-NiS_2_/CoS_2_ heterostructures.

As shown in Figure 2a and Supplementary Figure S4, undoped pure NiS_2_ nanomaterial shows regular crystal structure and smooth surface. With the increase in Co doping concentration, the regular crystal structure changes to a coral flower shape, and the wheat spike structure can be observed in the further enlarged image, which is conducive to the exposure of the catalytic active centers, thereby improving the catalytic efficiency [30,31]. When the Co content reaches up to 27%, the Co-NiS_2_/CoS_2_ heterostructures has the largest catalytic active region and the best electrocatalytic performance. As shown in Figure 2b, the Co-NiS_2_/CoS_2_ heterostructures presents a linear structure. Figure 2c is the HRTEM image of 27% Co doped Co-NiS_2_/CoS_2_ heterostructures. Obvious crystal segmentation lines were observed, forming ordered Co-NiS_2_/CoS_2_ heterostructures. The lattice constants of CoS_2_ and NiS_2_ are 0.248 nm (210) and 0.254 nm (210), respectively [26,31,32]. Figure 2e–g show EDXA (Energy dispersive X-ray) elemental mapping of Ni, Co and S, respectively. Additionally, the energy dispersive X-ray elemental mapping further reveals that the CoS_2_ nanosheet was successfully attached to the NiS_2_ nanosheet, forming a highly exposed interface [33].

**Figure 3:**
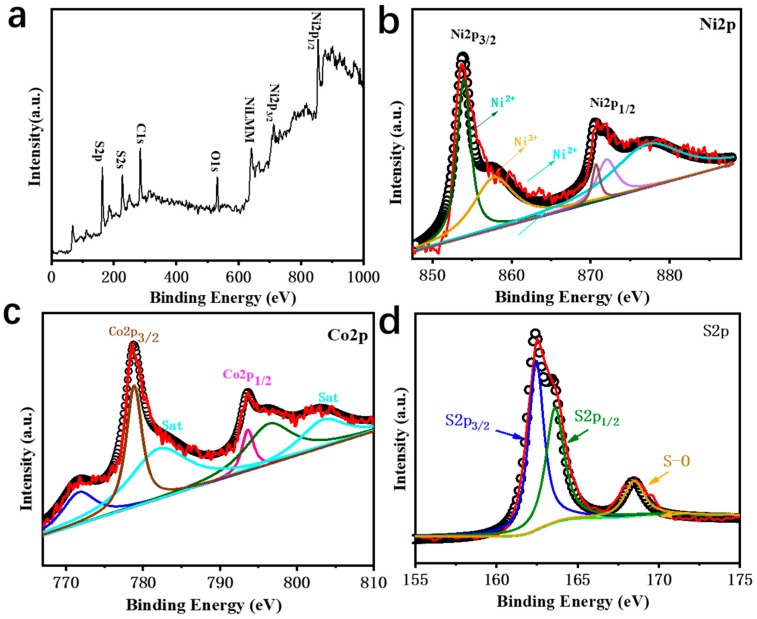
(**a**) Survey spectrum, and high-resolution; (**b**) Ni 2p; (**c**) Co 2p spectrum; (**d**) S 2p spectrum.

By means of EDXA and XPS (X-ray photoelectron spectroscopy), the chemical composition and valence state of Co-NiS_2_/CoS_2_ were further understood. Figure 3a shows the complete XPS spectrum of 27% Co-NiS_2_/CoS_2_ heterostructures. The peaks observed at 854.2 eV and 855.7 eV are attributed to Ni2p3/2 and Ni2p1/2 of Ni^2+^, respectively, and the peaks at 871.9 eV and 875.5 eV are attributed to Ni2p3/2 and Ni2p1/2 of Ni^3+^, respectively. The existence of NiS_2_ was further confirmed by the peaks of Ni^2+^ and Ni^3+^ and the satellite peaks of 861.7 eV and 879.6 eV (Figure 3b) [34,35]. As the XPS spectrum of Co is shown in Figure 3c. The Co 2p spectrum was deconvoluted to three spin–orbit doublets. The peaks at binding energies of 778.9 and 793.6 eV were assigned to Co of Co: NiS_2_, and those at 771.6 and 796.1 eV originated from Co:CoS_2_. Two shake-up satellites corresponding to oxidized Co species of Co:NiS_2_/CoS_2_ were also observed [33,36,37]. The peaks of S2p3/2 and S2p1/2 were observed at about 162.5 eV and 163.7 eV, respectively. Due to slight surface oxidation, the weak peak at approximately 168 eV can be identified as an S-O bond (Figure 3d) [31,38].

**Figure 4:**
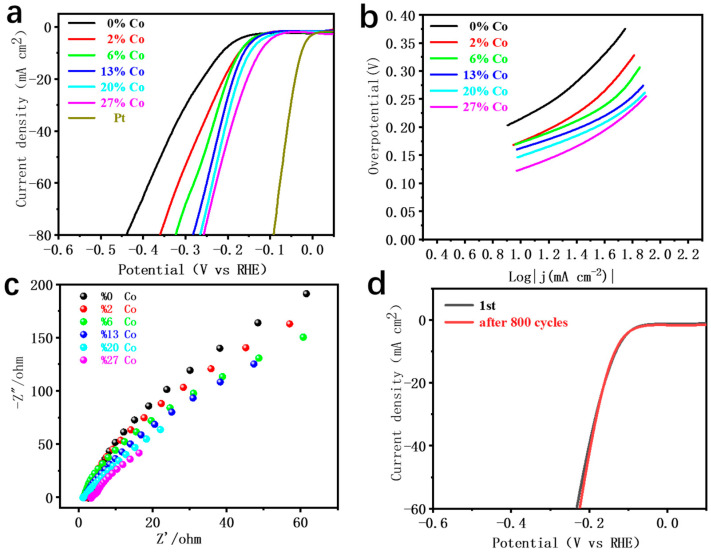
(**a**) LSV curves of Co-NiS_2_/CoS_2_ heterostructures with different Co doping concentrations in H_2_SO_4_ solution; (**b**) Tafel curves of Co-NiS_2_/CoS_2_ heterostructures with different Co doping concentrations in H_2_SO_4_ solution; (**c**) electrochemical impedance spectroscopy of samples; (**d**) LSV curves before and after 800 CV cycles.

Yang et al. [39] reported one 3D hybrid of CoSe nanoparticles encapsulated nitrogen-doped carbon nanotubes graft onto nitrogen-doped carbon nanosheets (denoted as CoSe@NCNT/NCN) prepared by a one-step method. Its overpotential in the 0.5 M H_2_SO_4_ solution is 197 mV, and the Tafel slope is 43 mV·dec^−1^. The electrode we prepared is based on NiS_2_. After Co doping; the catalytic performance of the formed NiS_2_/CoS_2_ heterostructure has been significantly improved as the amount of Co doped increases. In our experiment, we tested linear sweep voltammetry (LSV) to study the catalytic efficiency of Co-NiS_2_/CoS_2_ heterostructures with different Co doping ratio. The Co doping ratios in the heterostructures by precise weighing are 0%, 2%, 6%, 13%, 20% and 27%, respectively. As shown in Figure 4a, as the proportion of Co doping increases, the overpotential is −214.3, −173.3, −174, −162.3, −144.3 and −133.3 mV, respectively. In order to compare with the hydrogen evolution of precious metals, we further tested the LSV curve of the Pt electrode, and the overpotential of Pt was about −26.8 mV. With the increase in the Co doping concentration, the overpotential of 214.3 mV for undoped Co changes to 133.3 mV when the Co doping ratio is 27%. The absolute value of the overpotential is significantly reduced by about 40%, and the hydrogen evolution catalytic performance is significantly enhanced. As shown in Figure 4b, it can be observed that with the increase in Co doping concentration, the Tafel slope presents a downward trend, and the slope is nearly doubled (0% Co-240 mV·dec^−1^, 27% Co-123 mV·dec^−1^). By testing the electrochemical performance of 27% Co doped sample after 800 CV cycles, the absolute value of overpotential is only 3 mV different from that before cycling, indicating that the sample has good stability (Figure 4d). Through the experimental data, we found that when the Co doping concentration is 27%, the absolute value of overpotential is the smallest, the hydrogen evolution performance is the best, and it also has good stability. As shown in Figure S1, when Co is not doped or the doping concentration is low, the Raman spectrum has two higher peaks at 270 cm^−1^ and 480 cm^−1^ [40,41]. With the increase in doping concentration, especially 27% Co doping, the heterostructures is formed, the peak width increases and the crystallinity decreases. This is further confirmed by the scanning electron microscopy (SEM) images of each Co doping concentration in Figure S4. When the initial state of Co is not doped, the SEM spectrum is angular crystal structure, and the crystal is evenly distributed on the carbon fiber tube. When the proportion of Co doping increases, flowerlike particles appear on the regular crystal. When the proportion of Co doping reaches 27%, the regular crystal basically disappears, and only irregular coral flowerlike structure can be seen on the carbon fiber.

As shown in Figures S2a and S3a, in order to analyze the activity difference between Co-NiS_2_/CoS_2_ heterostructures and undoped NiS_2_, we measured different scanning rates (5, 10, 20, 30, 40, 50, 60, 70, 80, 90 and 100) by cyclic voltammetry (CV). As shown in Figures S2b and S3b, the electrochemical surface area (ECSA) of undoped and 27% doped Faraday double-layer capacitor (Cdl) is obtained. The catalytic performance of the working electrode is normalized to 1 cm^−2^ [24,42,43]. We applied the specific capacitance (20–60 μF cm^−2^) of 40 μF cm^−2^ here to calculate the ECSA [4]. The surface active area of the doped catalytic material is about 0.72 cm^−2^, and the surface active area of pure NiS_2_ is 0.61 cm^−2^.The calculated results show that the catalytic activity of the electrode doped with 27% Co is stronger, which indicates that the catalytic activity of the electrode doped with Co is enhanced. Figure 4c is the electrochemical impendence spectroscopy (EIS) diagram of the different doping concentrations. Further experimental studies show that with the increase in the Co doping ratio (34%), the structure and properties of the heterostructures are not significantly different from that of the heterostructures doped with 27%. As the amount of Co doping continues to increase, the surface of the catalyst is covered with a large number of particles, the nanowires are reduced, and the active area of the catalyst is reduced. A high proportion of Co doping cannot achieve a better doping income. The results show that with the increase in Co doping in a certain range, high doping concentration has better conductivity, and the catalytic performance is also improved compared with low doping concentration. 

## 4. Conclusions

To sum up, we prepared Co doped nanowires with different Co concentrations by hydrothermal method Co-NiS_2_/CoS_2_ heterostructures, and through experimental verification we found that Co doping can greatly improve the efficiency of transition metal sulfide electrocatalytic hydrogen production. At the same time, we found that the best catalytic efficiency is achieved when the doping concentration is 27%, which is expected to solve the problem of high cost and difficult large-scale industrial production of traditional noble metal catalytic hydrogen production, and provide a practical idea and method for large-scale production of hydrogen clean energy. 

## Data Availability

Data is contained within the article or Supplementary Materials.

## Supplementary Materials

The following are available online at https://www.mdpi.com/article/10.3390/nano11051245/s1, Figure S1: Raman spectra of Co doped Co-NiS_2_/CoS_2_ heterostructures with a-f ratios of 0%, 2%, 6%, 13%, 20% and 27%, respectively; Figure S2: (a) Cyclic voltammograms of 27% Co doped Co-NiS_2_/CoS_2_ heterostructures at different scan rates; (b) Calculation of the relationship between scanning rate and 27% Co doped Co-NiS_2_/CoS_2_ heterostructures in double-layer capacitor by linear fitting of capacitive current; Figure S3: (a) Cyclic voltammograms of 0% Co doped Co-NiS_2_/CoS_2_ heterostructures at different scan rates; (b) Calculation of the relationship between scanning rate and 0% Co doped Co-NiS_2_/CoS_2_ heterostructures in double-layer capacitor by linear fitting of capacitive current; Figure S4: SEM spectra of Co doped Co-NiS_2_/CoS_2_ heterostructures with a-f ratio of 0%, 2%, 6%, 13%, 20% and 27%, respectively; Table S1. The comparison of electrocatalytic performance of the latest and related research.
