# Supplementary material for: Effect of Co Doping on Electrocatalytic Performance of Co-NiS2/CoS2 Heterostructures
Source: Nanomaterials (Basel). 2021 May 8;11(5):1245. doi: 10.3390/nano11051245 (PMC8150845; doi:10.3390/nano11051245)
Supplement: Supplementary file 1 [file nanomaterials-11-01245-s001.zip › nanomaterials-1194316-SI.pdf]

Supplementary Information

Effect of Co Doping on Electrocatalytic Performance of Co-NiS_2/CoS_2 Heterostructures

Zehui Peng [1], Shuai Lou [2], Yuan Gao [1], Lijun Kong [1], Shancheng Yan [1,*], Ka Wang [3] and Haizeng Song [3]

[1] School of Geography and Biological Information, Nanjing University of Posts and Telecommunications, Nanjing 210023, China; 1019172222@njupt.edu.cn (Z.P.); 1218063835@njupt.edu.cn (Y.G.); 1020173018@njupt.edu.cn (L.K.)

[2] Department of Materials Science and Engineering, University of California, Berkeley, CA 94720, USA; slou@berkeley.edu

[3] Collaborative Innovation Center of Advanced Microstructures, Nanjing University, Nanjing 210093, China; 1217063729@njupt.edu.cn (K.W.); DG1823028@smail.nju.edu.cn (H.S.)

* Correspondence: yansc@njupt.edu.cn

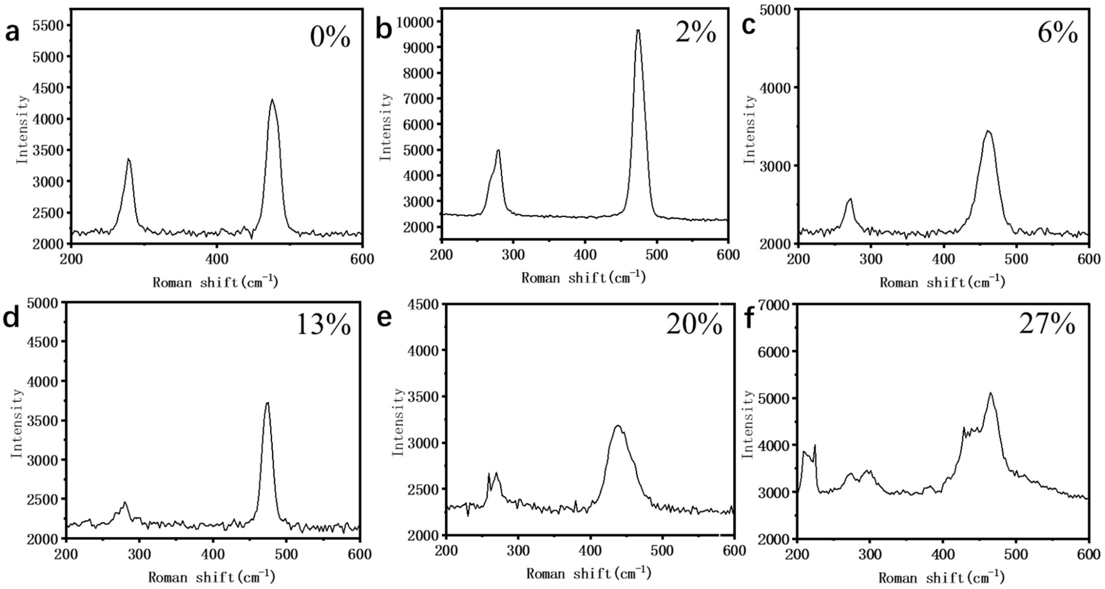

Figure S1. Raman spectra of Co doped Co-NiS_2/CoS_2 heterostructures with a-f ratios of 0%, 2%, 6%, 13%, 20% and 27%, respectively

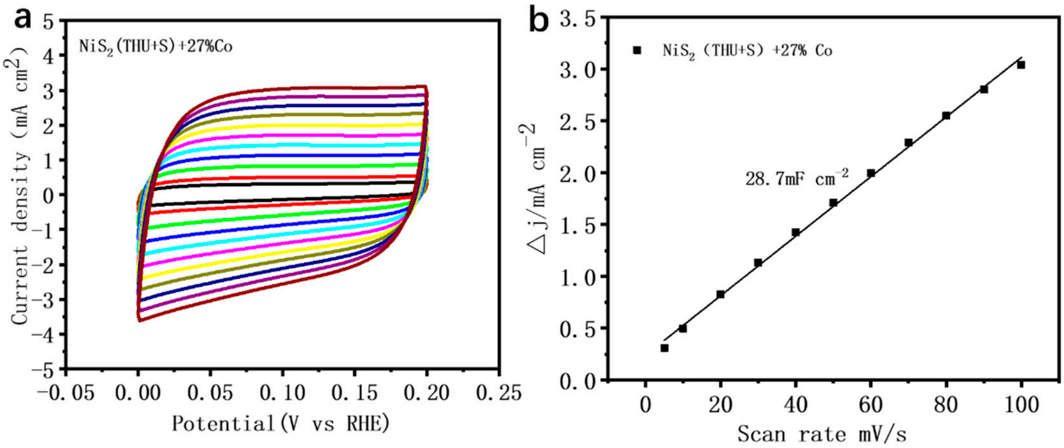

Figure S2. (**a**)Cyclic voltammograms of 27% Co doped Co-NiS_2/CoS_2 heterostructures at different scan rates; (**b**) Calculation of the relationship between scanning rate and 27% Co doped Co-NiS_2/CoS_2 heterostructures in double-layer capacitor by linear fitting of capacitive current

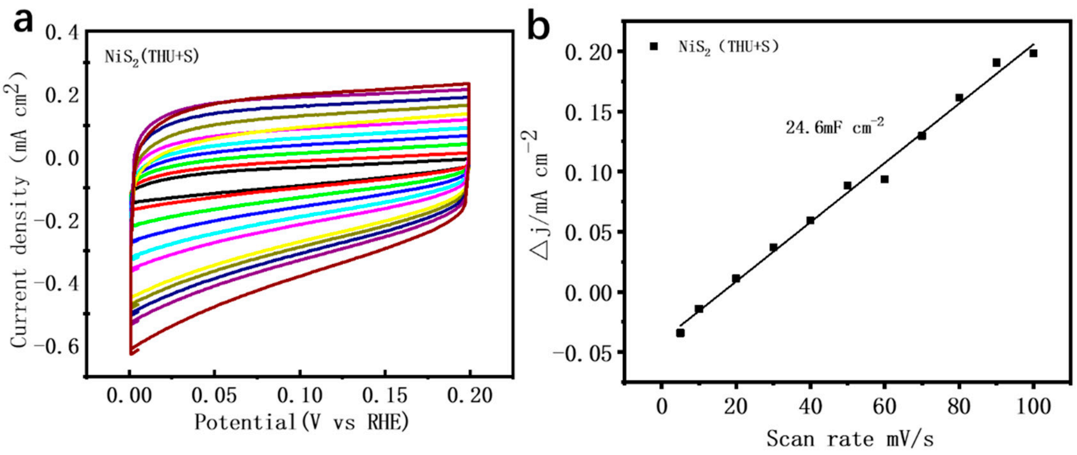

Figure S3. (a)Cyclic voltammograms of 0% Co doped Co-NiS_2/CoS_2 heterostructures at different scan rates;(b) Calculation of the relationship between scanning rate and 0% Co doped Co-NiS_2/CoS_2 heterostructures in double-layer capacitor by linear fitting of capacitive current

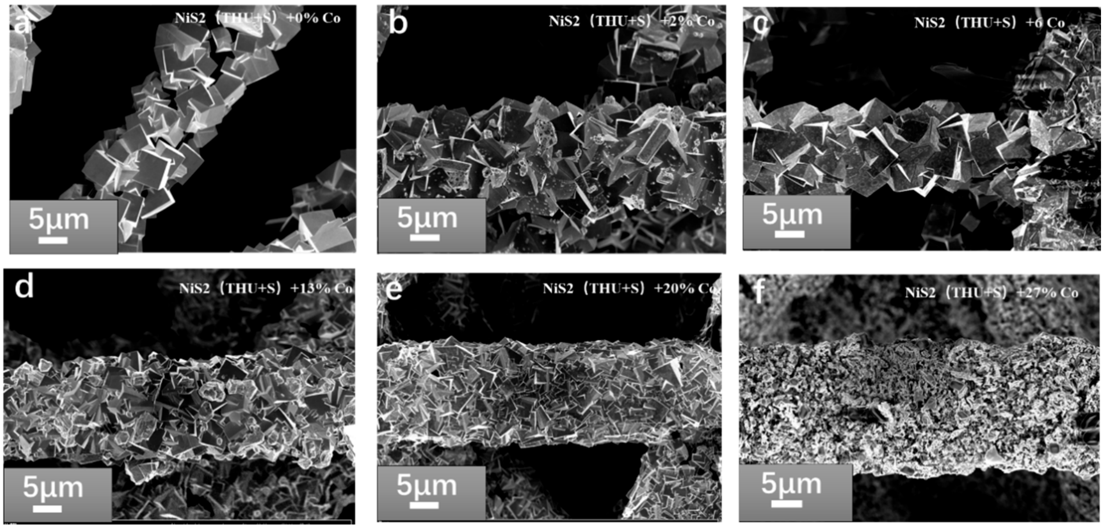

Figure S4. SEM spectra of Co doped Co-NiS_2/CoS_2 heterostructures with a-f ratio of 0%, 2%, 6%, 13%, 20% and 27%, respectively

Table S1. The comparison of electrocatalytic performance of the latest and related research.

Catalyst	Test solution	Overpotential （10mAcm^{-2}）	Tefel slope （mV·dec^{-1}）	Stability
**CoSe@NCNT/NCN[37]**	**0.5M H_2SO_4**	**197mV**	**43**	
**FeS nanosheets[4]**			**36.9**	
**Co-NiS_2/CoS_2**	**0.5M H_2SO_4**	**133mV**	**123**	**800CV**
**MoS_2[S1]**	**1 M KOH**	**234 mV**		
**Mo2C@CN[S2]**	**0.5M H_2SO_4**	**202 mV**	**69**	**2000 cycles in the acid media**
**CoNiP/NCNTs[S3]**	**0.5M H_2SO_4**	**75mV**		

References

S1. Wu, Z., Liu, X., Song, M., Zhao, Y. Mos2/cob with se doping on carbon cloth to drive overallwater splitting in alkaline electrolyte. *Sustainable Energy & Fuels*, **2020**, *4*, doi: 10.1039/D0SE00864H.

S2. Li, Yang.， Huang, Qilin.，Wu, Hongbiao.， Cai, Lun.， Du, Yiming.， Liu, Shiqiu.， Sheng, Zongqiang.， Wu, Mingzai. N-doped Mo2C nanoblock for efficient hydrogen evolution reaction [J]. *Journal of Solid State Electrochemistry*, **2019**, *23*，1-8，doi: 10.1007/s10008-019-04302-9.

S3. Xia, J., Li, S. M., Gao, S., Xie, S., Liu, H. Preparation of cobalt-based nanomaterials carried by nitrogen-doped carbon nanotubes as high performance electrocatalysts for hydrogen evolution reaction. *New Carbon Materials*, **2020**，*35*, 87-96，doi: 10.1016/S1872-5805(20)60477-2.
